# A dual-mechanism antimicrobial peptide with antimutagenic activity targets the replisome and induces cell envelope stress

**DOI:** 10.1128/msphere.00068-25

**Published:** 2025-08-29

**Authors:** Amanda Holstad Singleton, Olaug Elisabeth Torheim Bergum, Jana Scheffold, Synnøve Brandt Ræder, Lilja Brekke Thorfinnsdottir, Lisa Marie Røst, Caroline Krogh Søgaard, Per Bruheim, Marit Otterlei

**Affiliations:** 1 Department of Clinical and Molecular Medicine, Norwegian University of Science and Technology (NTNU) 8018, Trondheim, Norway; 2 Department of Biotechnology and Food Science, Norwegian University of Science and Technology (NTNU) 8018, Trondheim, Norway; University of Nebraska Medical Center College of Medicine, Omaha, Nebraska, USA

**Keywords:** APIM, β-clamp, cell-penetrating peptide, antimutagenic, transcriptomics, proteomics, *Escherichia coli*

## Abstract

To combat the growing threat of multidrug-resistant bacteria, we need to develop novel antibiotics with unique modes of action. This study investigates the antibacterial properties of BTP-001 toward *Escherichia coli*. BTP-001 targets the β-clamp subunit of the DNA Pol III holoenzyme and is composed of the binding motif APIM linked to a cell-penetrating part composed of 11 arginine residues (R11). Our data indicate that R11 facilitates energy-dependent transport of BTP-001 across the cell membrane, possibly via iron transport systems such as the TonB system. The full-length BTP-001 peptide rapidly disturbs membrane integrity, inducing expression and activation of the Cpx cell envelope stress response. This response likely triggers the production of reactive oxygen species (ROS), contributing to the rapid bactericidal effect, as evidenced by increased short-term survival by addition of a ROS scavenger. Furthermore, this study confirms that BTP-001 targets DNA replication and reduces resistance development by inhibiting translesion synthesis. In addition, our data suggest that the β-clamp is associated with ribosomal complexes and that BTP-001 disrupts translational processes. In conclusion, BTP-001 exhibits a multifaceted mode of action, which strengthens its potential as a novel therapeutic drug against antibiotic-resistant bacteria.

**IMPORTANCE:**

As antimicrobial resistance (AMR) increases, the world needs new antibiotics with new modes of action to avoid cross-resistance. In this study, we investigated how BTP-001, a novel cell-penetrating peptide that contains a protein-binding motif for the essential DNA replication protein β-clamp, kills bacteria. We demonstrate that BTP-001 has a dual mode of action in which it i) targets the β-clamp and inhibits replication and mutagenesis and ii) disrupts the bacterial cell envelope, causing ROS accumulation and rapid cell death. In addition, our data indicate that BTP-001 affects translation, suggesting that the β-clamp may have unknown roles beyond replication. Our data also suggest that the bacterial import of BTP-001, via the cell-penetrating part of the peptide, is dependent on active transport and involves iron uptake mechanisms. BTP-001 has many properties that could be useful for further development as a new antibiotic.

## INTRODUCTION

Increasing antimicrobial resistance (AMR) drives the need for novel antibiotics. Antimicrobial peptides (AMPs) are naturally produced by various organisms (e.g., plant and mammalian cells, yeast, and bacteria) as part of their defense mechanisms. While most AMPs are cationic and target the negatively charged bacterial membrane, some reach the cytoplasm and target intracellular components. A key characteristic of AMPs, which makes them attractive as next-generation antibiotics, is their potential for multiple targets. This multifaceted approach broadens their antimicrobial spectrum and reduces the likelihood of resistance development (1, 2). However, the limited stability and potential cytotoxicity of many linear AMPs reduce their therapeutic potential (3). To address these challenges, synthetic AMPs can be designed with optimized properties for therapeutic use. To facilitate uptake, synthetic peptides with intracellular targets are often linked to carrier molecules (e.g., cell-penetrating peptides). Among cell-penetrating peptides, cationic homopolymers, particularly polyarginine, have demonstrated efficient membrane permeabilization in bacteria (4). While the exact mechanism of translocation across bacterial membranes is unknown for arginine-rich cell-penetrating peptides, evidence supports that the transportation depends on the membrane potential and does not permanently damage the membrane (5–7).

The synthetic β-clamp targeting peptides (BTPs) contain the AlkB homolog 2 proliferating cell nuclear antigen (PCNA)-interacting motif (APIM) linked to a cell-penetrating peptide composed of 11 arginine residues (R11). We have previously shown that BTPs bind to the β-clamp and inhibit replication and translesion synthesis (TLS) (6). The current lead peptide, BTP-001, displays potent and rapid bactericidal activity against a broad range of gram-positive and gram-negative bacteria, including biofilm-producing bacteria and the highly concerning ESKAPE pathogens (*Enterococcus faecium*, *Staphylococcus aureus*, *Klebsiella pneumoniae*, *Acinetobacter baumannii*, *Pseudomonas aeruginosa*, and *Enterobacter* species) (6, 8, 9). Additionally, as TLS is inhibited, BTP-001 exhibits a reduced tendency for resistance development compared to conventional antibiotics, and with a novel mode of action (MoA), no cross-resistance has been observed (9). In addition to promising results in prosthetic joint and topical wound infections (8, 9), these findings highlight BTPs' potential as novel therapeutic candidates.

Omics technologies offer powerful tools to elucidate the MoA of antibiotics. Transcriptomics reveals changes in gene expression, including upregulated stress pathways. A subfield of proteomics selects for activated signaling proteins and protein complexes with exposed ATP-/GTP-binding motifs using the multiplexed kinase inhibitor bead (MIB) assay, which may complement transcriptomics by capturing not only changes in protein levels but also protein activation and deactivation (10–13). Previously, we demonstrated that BTP-001 affects proteins involved in replication, translation, redox sensing, and cell division in *S. aureus* (12). However, the precise mechanism underlying BTP-001’s rapid bactericidal effect remains elusive. In this study, we employ transcriptomics and a sub-proteomics approach to explore the response of *Escherichia coli* to BTP-001 at several time points. This combined approach aims to shed light on BTP-001’s MoA, including the uptake mechanism of R11 and potential non-canonical roles of the β-clamp.

## MATERIALS AND METHODS

### Bacterial strains and media

Unless otherwise noted, experiments were conducted with *E. coli* K-12 MG1655 cultivated in cation-adjusted Mueller-Hinton Broth II (CAMHB; Becton Dickinson, USA) or mineral media (MM). Specific experiments used *E. coli* K-12 BW25113 and the following Keio collection single-gene knockout mutants derived from *E. coli* K-12 BW25113: Δ*arcA*, Δ*degP*, Δ*cpxR*, Δ*cpxA,* Δ*tonB*, and Δ*exbB* (14). The MM was prepared with glucose (4 g/L) as the carbon source and NH_4_Cl (5 g/L) as the nitrogen source, as described in (15). Pre-cultures were prepared by inoculating the *E. coli* glycerol stock in CAMHB or MM with incubation for 16 ± 1 h (37°C, 250 rpm).

### Antibiotic compounds

BTP-001 (Innovagen, Sweden and Biosynth, Netherlands) consists of the APIM motif (in bold) connected to an arginine tail via a linker and has an acetylated N-terminus and an amidated C-terminus: Ac-MD-RWLVK-GILQWRKI-R11-NH_2_ (6). BTP-001A (Innovagen) contains the motif RALVK connected to an arginine tail via the same linker as BTP-001. This mutation in APIM, W2A, reduces the affinity for the β-clamp (6). The cell-penetrating peptides R11 (Innovagen) consist of 11 arginine residues: Ac-RRRRRRRRRRR-NH_2_. Lyophilized peptides were dissolved in water to stock solutions of 1–10 mM (based on weight) and stored at 4°C. All concentrations are given as net peptide concentrations.

### Minimal inhibitory concentration (MIC) assay

MIC determination was performed according to standard susceptibility testing established by the Clinical and Laboratory Standards Institute guidelines (16), with modifications as described earlier (6). Briefly, the bacterial suspension in CAMHB was adjusted to 0.5 McFarland (McF) and diluted to 5 × 10^5^ CFU/mL. Peptides were added as 10% of the total volume. Microtiter plates were inspected for visible growth after incubation (24 h, 37°C). Previously published MIC values for BTP-001, BTP-001A and R11 are given in Table 1

**TABLE 1:** MIC in E. coli MG1655 in cation-adjusted Mueller-Hinton broth II (CAMHB)^*a*^

Peptide/drug	MIC (μM)
BTP-001	3-4
BTP-001A	9
R11	14.5
Ciprofloxacin	0.05

^*a*^ Values are from references 6, 13.

For MIC supplemented with iron, FeSO_4_ (80 µM) was added to CAMHB and MM. ZnSO_4_ (80 µM) was used as an ionic control to ensure that any microbial inhibition observed was due to the unique properties of Fe²⁺ rather than a general effect of divalent cations. MIC with a reactive oxygen species (ROS) scavenger was conducted with CAMHB supplemented with dimethyl sulfoxide (DMSO; 7.5% [vol/vol]).

### Serial passage

Ciprofloxacin was tested alone and in combination with BTP-001 or BTP-001A. Serial twofold dilutions of ciprofloxacin, with or without a fixed concentration of 4 µM BTP-001 or BTP-001A, were prepared in 96-well round-bottom plates. In the first passage, ciprofloxacin concentrations ranged from 0.004 to 0.016 µg/mL and were gradually increased as resistance developed, while the BTP-001 and BTP-001A concentrations remained constant at 4 µM. The experiment was conducted over 14 consecutive days to assess resistance development.

On day 1, an overnight *E. coli* culture was diluted to 5 × 10⁵ CFU/mL and added to each well containing ciprofloxacin alone or combined with 4 µM of either BTP-001 or BTP-001A. After a 24 hour incubation, the MIC was determined by visual inspection of bacterial growth. For each passage, the culture from the well containing 0.5× MIC was diluted 1:100 and transferred into a new 96-well plate with fresh media and twofold ciprofloxacin dilutions (0.5×, 1×, 2×, and 4 × MIC) alone or with 4 µM BTP-001 or BTP-001A. This process was repeated daily for 14 days.

### ROS assay

ROS production was measured using the cell-permeable probe 2′,7′-dichlorofluorescein diacetate (DCFH-DA; Sigma-Aldrich, USA). The pre-culture was prepared in MM and grown overnight, diluted to OD_600_ = 0.0125 in MM, and incubated to OD_600_ = 0.2 (37 °C, 250 rpm). A 96-well plate was prepared with DCFH-DA (5 µM). Designated wells, in six replicates, were treated with varying concentrations of BTP-001 and R11. Positive control wells were treated with H_2_O_2_ (10 mM), while untreated control wells received no treatment. The bacterial culture was added to the plate to a total volume of 200  µL in each well. The plate was incubated in the dark for 30  min (37°C, 400  rpm). Fluorescence was measured in a microplate reader (FluoSTAR Omega; BMG Labtech, Germany), with excitation spectra at 485  nm and emission spectra at 520  nm. All experiments were performed as three biological replicates. A two-sided Student’s *t*-test was used to determine the *P*-value.

### Time-kill assay

Pre-cultures of *E. coli* MG1665, *E. coli* BW25113, and the Keio collection mutants Δ*arcA*, Δ*degP*, Δ*cpxR*, and Δ*cpxA* were adjusted to 0.5 McFarland (1 × 10^8^ CFU/mL) and diluted to 5 × 10^5^ CFU/mL in CAMBHFor experiments studying the effect of ROS, bacteria were pretreated with DMSO (5% and 7.5% [vol/vol]). A 96-well plate was prepared with BTP-001 (1.33 µM or 4 µM), while phosphate-buffered saline (PBS) was added to untreated control wells. The bacterial culture was added to the plate to a total volume of 200  µL in each well. The plate was incubated at 37°C and 250 rpm. At 0, 15, 30, 45, and 90 min post-treatment, an appropriately diluted bacterial suspension was plated on Luria-Bertani (LB) agar plates. The agar plates were incubated for 16 ± 1 h at 37°C, and CFU/mL was calculated. All experiments were performed as three biological replicates. A two-sided Student’s *t*-test was used to determine the *P*-value.

### Survival assay (CFU/mL)

Pre-cultures of *E. coli* MG1655 were diluted to an OD_600_ of 0.233 in PBS and transferred to protein low-bind tubes. The bacterial cultures were then treated with 4 µM BTP-001, or water as a control, on ice or at 37°C with gentle agitation. After 10 min of treatment, preheated fetal bovine serum was added to a final concentration of 50% (vol/vol) and incubated for 2 minutes at 37°C to inactivate BTP-001. The bacterial cells were then pelleted (5,000 rcf, 10 min, RT), resuspended in 500 µL PBS, and incubated at 37°C for 30 minutes before plating a serial dilution on LB agar plates for CFU/mL determination.

### *In vitro* translation assay

The effects of BTP-001, BTP-001A, and R11 on translation were assessed using the *E. coli* extract system for circular DNA (Promega, L1020). The assay was performed according to the manufacturer’s instructions and as described in (17) with minor changes. Briefly, the 25 µL reactions were carried out in Eppendorf tubes with 1 µL luciferase control RNA (Promega, L4561) and incubated at 37°C for 1 h. After stopping the reaction for 5 min in an ice bath, 25 µL of Luciferase Assay Reagent (Promega, E1483) was added to each reaction. Twenty microliters of each reaction was then added to 50 µL Luciferase Assay Reagent in an opaque 96-well plate, and luminescence was measured using a microplate reader (FluoSTAR Omega; BMG Labtech, Germany) for 10 min at 37°C.

### Omics sampling from batch cultivation

#### Batch setup

The batch setup was prepared using 1 L bioreactors (Applikon, Netherlands) with 1 L CAMHB. The pH probe was calibrated with pre-mixed solutions of pH 4 and pH 7. pH 7 was maintained by automatic titration of 3 M HCl and 4 M NaOH. The dissolved oxygen (DO) probe was calibrated to 100% DO in the bioreactor after the CAMHB reached 37°C with an aeration rate of 500 mL min^−1^ and 200 rpm stirring. The DO probe was flushed with nitrogen gas before use to ensure electrode sensitivity. Stirring was adjusted between 200 and 600 rpm to maintain a DO above 40%. The inflow air was sterile-filtered using a 0.2 µm filter. O_2_ consumption and CO_2_ production were constantly monitored by analyzing the off-gas with the Prima Bench Top Process Mass Spectrometer gas analyzer (Thermo Fisher Scientific, USA). Excess foam formation was prevented by manually adding a silicone polymer antifoam (Sigma-Aldrich).

#### Treatment

The omics experiments were performed as two independent treatment setups in bioreactors. The omics data from the first treatment setup were used to decide relevant antibiotic concentrations and sampling time points to include in the second setup. The first treatment setup consisted of BTP-001 (1 µM) and an untreated control, while the second setup consisted of BTP-001 (3 µM and 4.5 µM), R11 (1 µM), and an untreated control. The *E. coli* MG1655 pre-culture was diluted to OD_600_ = 0.0125 in the bioreactors. Treatment with BTP-001 or R11 was performed at OD_600_ = 0.2, while one batch culture was left as the untreated control. The experiments were performed as three biological replicates. An overview of the treatments is shown in Table 2.

**TABLE 2:** An overview of omics experiments^*b*^ performed from batch cultivation^*a*^

Treatment	Media	OD_600_	Time point (min)	Transcriptomics	MIB assay
BTP-001 (1 µM)	CAMHB	0.2	−1	X	
			1	X	X
			10	X	X
			25	X	X
			50	X	X
			75	X	
			120	X	X
BTP-001 (3 µM)	CAMHB	0.2	10	X	X
BTP-001 (4.5 µM)	CAMHB	0.2	10	X	
R11 (1 µM)	CAMHB	0.2	10	X	X

^*a*^ X indicate experiments performed.

^*b*^ The *E. coli* culture was grown in cation-adjusted Mueller-Hinton broth II (CAMHB) and received treatment at OD_600_ = 0.2. Sampling for transcriptomics and the multiplexed kinase inhibitor bead (MIB) assay are shown as post-treatment time points (min).

#### Sampling for transcriptomics and the multiplexed kinase inhibitor bead (MIB) assay

For treatment with 1 µM BTP-001, sampling from the bioreactor was performed 1 min before treatment (approximately at OD_600_ = 0.19) and 1, 10, 25, 50, 75, and 120 min post-treatment. For treatment with 4.5 µM BTP-001, 3 µM BTP-001, and 1 µM R11, sampling from the bioreactor was performed 10 min post-treatment. At the designated time points, samples were taken for transcriptomics and the MIB assay with one technical replicate per biological replicate.

For the MIB assay, a volume of *E. coli* culture between 2 and 10 mL (based on the OD_600_ at the sampling timepoint) was centrifuged (4,500 rcf, 10 min, 4°C). The supernatant was discarded, and the pellet was snap-frozen in N_2_ (*l*) and stored at −80°C until further processing.

For transcriptomics, sampling was performed according to the protocol provided with the RNeasy Mini kit (Qiagen, Germany). The sampled volume was based on the OD_600_ at each sampling time point to ensure approximately 3.35 × 10^8^ cells per sample. The sample was immediately added to two volumes of RNA protect and vortexed. After a minimum of 5 min, the samples were centrifuged (4,500 rcf, 10 min). The supernatant was discarded, and the pellet was snap-frozen in N_2_ (*l*) and stored at −80°C until further processing.

### Proteomics using the MIB assay

#### Sample processing and analysis

Cell extracts were prepared by a combination of lysozyme treatment (1 mg/mL) and freeze-thaw cycling between N_2_ (*l*) and H_2_O (37°C). The MIB assay with on-column trypsinization was used to isolate ATP-/GTP-binding proteins from 100 µL cell extract containing 1 mg/mL protein (11). The kinase inhibitor mix used for coupling to the Sepharose beads was previously optimized for maximum pull-down of the bacterial proteome (12). The optimized mix consisted of an equal mix of the kinase inhibitors purvalanol B (Tocris, UK), L-1, and L-3 (made in-house). Samples were stored at −20°C until analysis.

Sample analysis was performed by the Proteomics Core Facility at NTNU using liquid chromatography (LC) MS/MS. A timsTOF Pro 2 (Bruker Daltonics, USA) connected to a nanoElute (Bruker Daltonics) HPLC system was used to perform the LC-MS/MS analysis. Peptide separation was conducted using a Bruker PepSep column (25 cm x 75 µm x 1.5 µm) kept at 50°C. The LC used running buffers A (0.1% formic acid) and B (0.1% formic acid in acetonitrile) with a gradient ranging from 2% B to 40% B over 40 minutes at a flow rate of 250 nL/min. Subsequently, within 1 min, the gradient transitioned to 95% B and a flow rate of 300 nL/min, which was maintained for 9 min. The MS instrument was operated in the data-dependent acquisition parallel accumulation serial fragmentation (DDA-PASEF) mode with 10 PASEF scans per acquisition cycle and accumulation and ramp times of 100 ms each. The “target value” was set to 20,000, and dynamic exclusion was activated and set to 0.4 min. The quadrupole isolation width was set to 2 Th for *m/z* < 700 and 3 Th for *m/z* > 800.

#### Data analysis

MS data were processed using MaxQuant v 2.1.3.0 for label-free quantification (LFQ) of proteins (18). The following search parameters were used: the digestion enzyme was specified as trypsin with a maximum of two missing cleavages, variable modifications were set to oxidation (M), acetylation of protein N-terminal, and deamination (NQ), and fixed modifications were set to carbamidomethyl (C). LFQ min. ratio count was set to 1. Samples were queried against the imported *E. coli* K-12 reference proteome (including isoforms) downloaded from the UniProt website (https://www.uniprot.org/proteomes/UP000000625, accessed on 6 April 2022) and Andromeda, MaxQuant’s internal contaminants database. FDR for protein and peptide identification was set to 1%. Only unique peptides were used for definite protein group identification. The area under the peak curve was integrated to obtain peak abundances. The total abundance of all peptides identified for each protein during each run was used to normalize the abundance in every protein group using the LFQ algorithm (19) with minimum peptides ≥ 1.

LFQ values were analyzed in R v 4.1.2 using the DEP package v 1.18.0 from Bioconductor (20). The data were normalized using variance stabilizing transformation. Each time point was analyzed separately. Proteins identified in at least two biological replicates for either the treatment or control samples were included in the analysis. Missing values for proteins showing a similar trend of being upregulated (≥ 0) or downregulated (≤ 0) after treatment were imputed by random draws from a Gaussian distribution centered around a minimal value (“MinProb”) as MNAR (missing not at random), while the remaining missing values were imputed by the k-nearest neighbor method (“knn”) as MAR (missing at random). DEP uses protein-wise linear models combined with empirical Bayes statistics to find differentially enriched proteins. Differentially enriched proteins were defined with the Wilcoxon signed-rank test combined with *P*-value < 0.1 and presented as log_2_ fold change compared to the control.

A gene set enrichment analysis (GSEA) was performed to find significant GO terms. The input was a ranked list of all proteins based on their log_2_ fold change values at each time point and for each treatment group. The function gseGO() was used to perform the GSEA with a *P*-value cutoff set to 0.05. The org.EcK12.eg.db v 3.18.0 annotation database was used.

### Immunoprecipitation and His-tag pull-down assay

The immunoprecipitation (IP) and His-tag pull-down assays were conducted as described in (12). Briefly, *E. coli* BL21 cells containing plasmids for expression of the EYFP-tagged APIM-peptide or His-tagged β-clamp (pET28/pET16b, respectively) were induced with isopropyl β-d-1-thiogalactopyranoside (IPTG; 300  nM). *E. coli* BL21 expressing only EYFP and only His-tag were used as controls. IP with Dynabeads Protein A (4.5  mg, Invitrogen, USA) and α-GFP antibody (Abcam, UK; ab290) was conducted with 0.5 mg cell extract from *E. coli* containing the EYFP-tagged APIM-peptide or just EYFP. TALON Metal Affinity Resins (Clontech Laboratories, Inc., USA) were used to pull down the His-tagged β-clamp and His-tag only control. The pelleted beads and resin collected from IP and His-tag purification, respectively, were prepared for MS analysis by trypsination and desalting with C18 stage tips. Overexpression of APIM fused to EYFP in *E. coli* BL21 has larger growth inhibitory activities than overexpression of EYFP only, even though the levels of protein expressions are significantly lower for EYFP only (data not shown) (6).

The MS data were processed in MaxQuant, as described above in "Data analysis" in “Proteomics using the MIB assay.” LFQ values were log_2_ transformed, and the mean values for the EYFP- and His-tag-only controls were subtracted from the EYFP-tagged APIM-peptide or His-tagged β-clamp, respectively, to yield the log_2_ fold-change for each protein. A two-sided Student’s *t*-test was used to calculate the *P*-value. Missing values were assumed to be missing not at random and imputed using the quantile regression imputation of left-censored data (QRILC) method implemented in the imputeLCMD R package (v 2.1). The QRILC approach imputes missing data from a left-shifted Gaussian distribution estimated via quantile regression.

### Transcriptomics

#### Sample processing and analysis

RNA was isolated using the RNeasy Mini Kit (Qiagen), according to the manufacturer’s protocol 4 (enzymatic lysis and proteinase K digestion of bacteria) and protocol 7 (purification of total RNA) with on-column DNase (Qiagen) digestion. The RNA concentration in each sample was measured using a NanoDrop-1000 spectrophotometer (Thermo Fisher Scientific). Samples were stored at −80°C until analysis.

Sequencing was conducted by the Genomics Core Facility at NTNU. RNA-sequencing libraries were prepared using the QIAseq FastSelect 5S/16S/23S kit (Qiagen) for rRNA removal and the QIAseq stranded RNA Lib kit (Qiagen) for library construction, according to the manufacturer’s instructions.

Briefly, 500 ng total RNA was used as the starting material. Removal of ribosomal RNA (rRNA) was conducted by a combined heat fragmentation (89°C for 7 min) and FastSelect hybridization protocol (75.4°C ramping process) where the FastSelect reagent inhibited reverse transcription of bacterial rRNA. Next, purification was conducted using QIAseq Beads, followed by a first-strand synthesis using an RNase H-Reverse Transcriptase (RT) in combination with random primers, a second-strand synthesis, end-repair, A-addition, and adapter ligation. The second-strand synthesis was performed using 5′ phosphorylated random primers, which enable subsequent strand-specific ligation. DNA fragments were further enriched by CleanStart library amplification (15 cycles of PCR). Finally, the libraries were purified using the QIAseq Beads, quantitated by qPCR using the Collibri Library Quantification Kit (Thermo Fisher Scientific), and validated using PerkinElmer DNA 1K/12K/Hi Sensitivity Assay LabChip on a Labchip GX instrument (PerkinElmer, USA). The size range of the DNA fragments was measured to be in the range of 270 to 570 bp and peaked around 355 bp.

Prior to sequencing, libraries were normalized and pooled to 2.3 pM and subjected to clustering on three NextSeq 500 HO flow cells (Illumina, USA). Finally, single-read sequencing was performed for 75 cycles on a NextSeq 500 instrument (Illumina), according to the manufacturer’s instructions. Base-calling was done on the NextSeq 500 instrument by RTA v 2.4.6. FASTQ files were generated using bcl2fastq2 Conversion Software v 2.20.0.422 (Illumina).

#### Data analysis

Processing of the sequence data was done with the ProkSeq v 2.0 (21) program using Docker, with the *E. coli* K-12 MG1655 reference genome ASM584v2 (RefSeq: GCF_000005845.2) and corresponding reference transcriptome. ProkSeq is a Docker-based, full RNA-Seq pipeline for prokaryotes, which includes quality assessment, alignment, gene counting, and analysis. The count tables countFile.csv, countFileNucleotideAvgCount.csv, and countFile_TPM_CPM.tsv were exported from ProkSeq and used for further analysis.

Count files were analyzed in R using the quasi-likelihood F-test in EdgeR v 3.36.0 from Bioconductor (22). The FDRs were calculated using the Benjamini-Hochberg procedure. Poorly expressed genes were filtered out, and library size was normalized using trimmed mean of M-value (TMM). Differentially expressed genes were defined with FDR < 0.05 and presented as log_2_ fold change compared to the control.

Hierarchical clustering of the time-course gene expression data was conducted using the hclust function in base R. Over enrichment analysis (ORA) of GO terms in the individual clusters was conducted using the Bioconductor package clusterProfiler v 4.10.0 and the function enrichGO(). The input was a list of DEGs from each cluster with at least an abs(log_2_ fold change) >0.5 during the time course. The *P*-value cutoff was set to 0.05, and the *q*-value cutoff was set to 0.2. For the high-dose BTP-001 and R11 analysis, which were only sampled at 10 min, a GSEA was performed to find significant GO terms. The input was a ranked list of all genes based on their log_2_ fold change values. The function gseGO() was used to perform the GSEA with a *P*-value cutoff set to 0.05. The org.EcK12.eg.db v 3.18.0 annotation database was used for both ORA and GSEA analyses.

#### Validation of RNA-seq by RT-qPCR

RNA-Seq data were validated using RT-qPCR. The gene *murB* was used as a reference gene, which had stable expression across the different treatments, based on RNA-seq data.

Reverse transcription of RNA to cDNA was performed using a high-capacity cDNA reverse transcription kit (Applied Biosystems, USA) with 1 µg RNA per 20 µL reaction, according to the manufacturer’s protocol. SYBR Green PCR Master Mix (Applied Biosystems) was used for performing qPCR. Each 10 µL reaction contained 100 ng template and 200 nM primer. A final concentration of 400 nM was used for *spy* and *cpxP*. Thermal cycling was performed on a T100 Thermal Cycler (Bio-Rad, USA) including a 10 min hot activation of the AmpliTaq Gold polymerase (Applied Biosystems) at 95°C, followed by 40 cycles of denaturation at 95°C and annealing/extension at temperatures from 56 to 68°C. The annealing temperature for each primer pair was optimized by performing a temperature gradient qPCR. The annealing temperatures as well as the primer pair sequences can be found in Table 3. Fluorescence was measured continuously, and the baseline adjustment of the Biorad T100 Thermal Cycler was used to determine the Ct in each reaction. After each qPCR, a melting curve experiment was conducted to confirm the presence of a single product peak. All reactions were performed as three biological replicates with three technical replicates per biological replicate.

**TABLE 3:** Primer sequences and annealing temperatures used for RT-qPCR

Gene	Forward primer	Reverse primer	Annealing temperature (°C)
*degP*	GCTCAGGTGGGTACTATGCC	CATCTCAGCGCCTTCAATGC	65
*ompF*	AGGCTTTGGTATCGTTGGTG	TTGTTCGCGTCGTACTTCAG	65
*spy*	AGCGTGACCAGATGAAACGT	TTGCTTTTTCTGTTCCGGCG	68
*nrdE*	ATGACGAACTGGTTGCCGAT	GGCGCTGTTAACCTGCAAAA	56
*cpxP*	TGGAGACAATGCATCGCCTT	TTTGGTTGCGGACTTTTGCC	68

## RESULTS AND DISCUSSION

### Experimental design and growth kinetics

The current lead peptide, BTP-001, consists of APIM (RWLVK) conjugated to R11 via a linker. While APIM has been shown to interact with the β-clamp and inhibit replication, BTP-001’s rapid bactericidal effect (reduced ability to form colonies in time-kill assays after 5 minutes), even in stationary phase cells, suggests additional targets outside replication (unpublished data; 8). If BTP-001 only inhibited DNA replication, the bactericidal effect would most likely be slower. Furthermore, arginine homopeptides, including R11, have demonstrated antibacterial properties at higher concentrations (6, 23). Therefore, we hypothesize that the rapid bactericidal effect of BTP-001 is due to APIM disrupting noncanonical roles of the β-clamp and/or unique effects of the full-length peptide composed of APIM–linker–R11.

**Fig 1:**
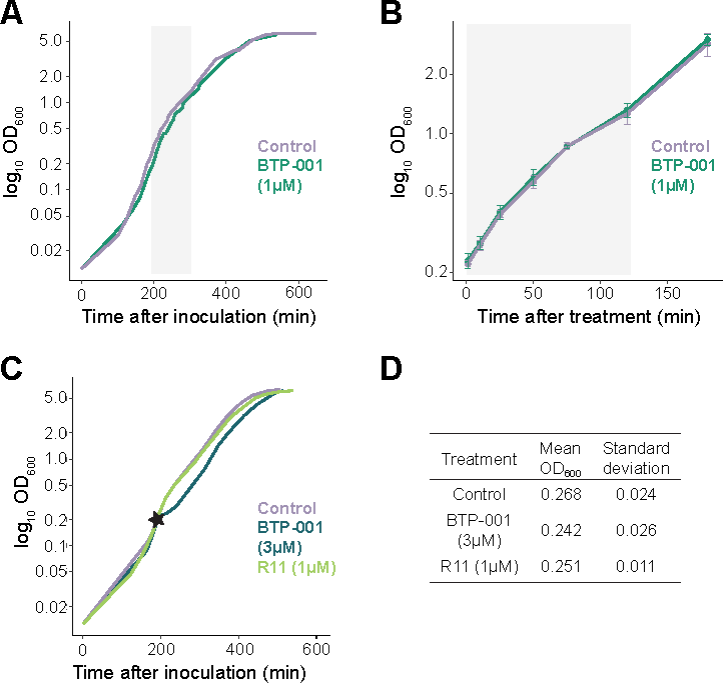
Growth kinetics of *E. coli* MG1655 treated with BTP-001 and R11. (**A**) The log_10_ OD_600_ from the time of inoculation until the stationary phase for the untreated control and BTP-001 (1 µM) in biological replicate (BR) 1. The growth curve is representative of all BRs. (**B**) The log_10_ OD_600_ for all BRs at the sampling time points for BTP-001 (1 µM) and the control. (**A, B**) The gray area indicates the sampling period. (**C**) The log_10_ OD_600_ from the time of inoculation until the stationary phase for the control and BTP-001 (3 µM) and R11 (1 µM) in BR1. The star indicates the treatment time. (**D**) The mean OD_600_ and standard deviation 10 min after treatment with BTP-001 (3 µM) and R11 (1 µM) for all BRs.

To elucidate BTP-001’s MoA, we conducted RNA-sequencing (RNA-seq) and used the MIB assay to examine *E. coli* MG1655 cultures treated with two doses of BTP-001 and one dose of R11. We chose a low dose of BTP-001 (1 µM; Fig. 1A and B) with no impact on growth and a high dose (3 µM; equivalent to MIC) that affected the growth (Fig. 1C) but did not yield extensive lethality during batch cultivation in bioreactors. A higher dose of BTP-001 (4.5 µM) was also tested, but the lethality was too high to obtain high-quality samples for further analysis. The R11 dose (1 µM) reflects the cell-penetrating part in the BTP-001 low dose. The high dose (3 µM) stalled growth for around 10 min before the culture recovered and eventually reached the same growth rate as the control (Fig. 1C). All treatment groups reached the stationary phase at an optical density (OD; 600 nm) approx. equal to 5 (Fig. 1A and C).

All experiments were conducted in bioreactors with continuous dissolved oxygen and off-gas analysis. This provided highly controlled conditions that yielded little variation between the biological replicates (Fig. 1B and D). All treatments were given at OD_600_ = 0.2 to ensure exponential growth during the entire sampling period (Fig. 1A, gray area). To investigate the temporal effects of BTP-001, the low dose of BTP-001 (1 µM) culture was sampled for transcriptomics and proteomics over a time course between 1 and 120 min after treatment (Fig. 1B). The high dose of BTP-001 (3 µM) and R11 (1 µM) was sampled for transcriptomics and proteomics 10 min after treatment.

### BTP-001 induces rapid expression of genes involved in membrane-associated processes

**Fig 2:**
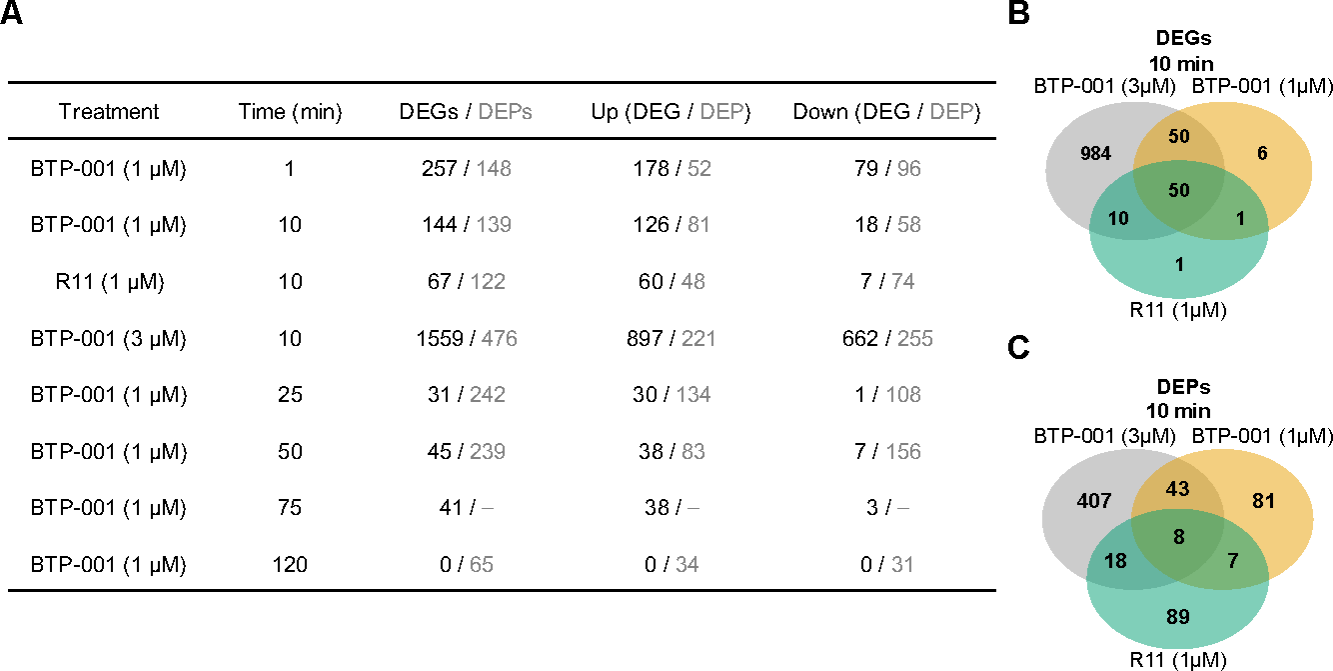
(**A**) Differentially expressed genes (DEGs) and differentially enriched proteins (DEPs) in *E. coli* following treatment with BTP-001 (1 and 3 µM) and R11 (1 µM) during exponential growth. FDR < 0.05 (DEG) and Wilcoxon signed-rank test combined with *P*-value < 0.1 (DEP). (**B**) Venn diagram of DEGs at 10 min. (**C**) Venn diagram of DEPs at 10 min.

Differentially expressed genes (DEGs) were identified by mapping reads to the *E. coli* K-12 MG1655 reference genome and comparing counts per gene to the untreated control. The analysis revealed differential expression (FDR < 0.05) starting 1 min after treatment with the low dose of BTP-001 (1 µM; Fig. 2A). The number of DEGs progressively decreased with increasing treatment time. R11 (1 µM) generated fewer (~25%) DEGs than BTP-001 (1 µM) and yielded only one unique DEG (Fig. 2A). The high dose of BTP-001 (3 µM), which was only sampled at 10 min, yielded 1,559 DEGs, 984 of which were unique. This reflects the stronger effect and greater stress response compared to the lower dose. Across all time points and treatments, the number of upregulated DEGs was higher than the number of downregulated DEGs. The 50 unique DEGs that resulted from both BTP-001 treatments but not R11 treatment included genes involved in cell envelope stress and cysteine catabolism (Fig. 2B).

As discussed above, the MIB assay enriches for proteins with an accessible ATP-/GTP-binding pocket, which is often considered to be indicative of an active form, or proteins in complex with the activated proteins (10–13). This method offers an advantage over whole-proteome analysis, which only provides a static snapshot of the total protein pool and prioritizes the most abundant proteins, by revealing dynamic changes in protein activation and including less abundant proteins. These changes can be protein activation, deactivation, or degradation. In contrast to the DEGs, the number of differentially enriched proteins (DEPs) increased until 25 min, and they were even detected 120 min after treatment with the low dose of BTP-001 (Fig. 2A). Additionally, the number of proteins with reduced enrichment following treatment was generally higher than those with increased enrichment, indicating deactivation, degradation, or inhibition of translation. The 43 DEPs that were only detected with BTP-001 treatment (Fig. 2C) included proteins involved in stress and membrane processes such as ATP synthesis. Unique R11 DEPs included proteins involved in iron homeostasis. Significantly more DEPs were identified following treatment with the higher dose of BTP-001 (3 µM) at 10 min, including downregulation of translational processes, as discussed below.

**Fig 3:**
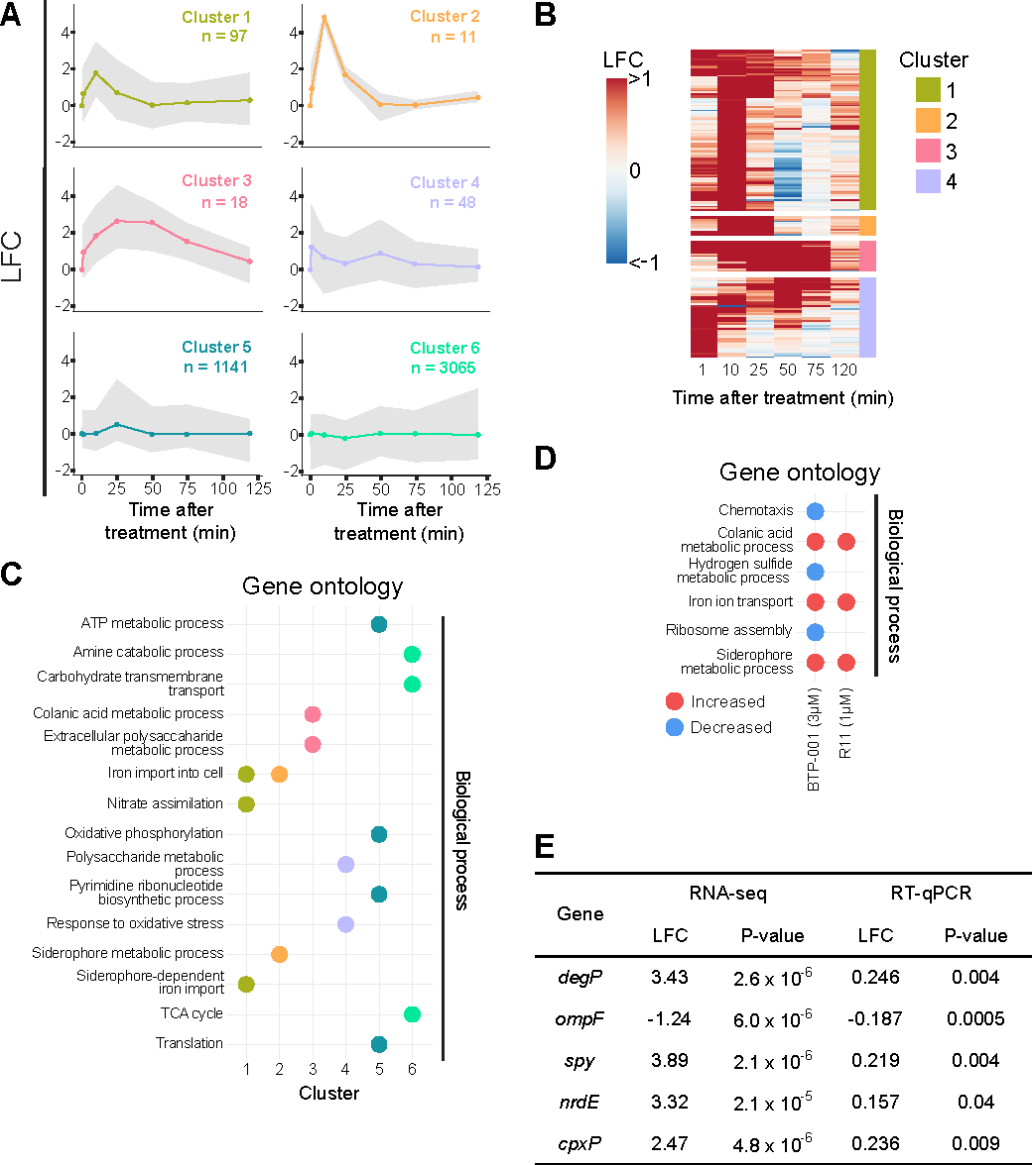
Overview of gene expression data for BTP-001- and R11-treated *E. coli.* (**A**) Log_2_ fold-change (LFC) expression profiles after BTP-001 (1 µM) treatment. Clusters based on hierarchical clustering of the expression pattern during the time course. (**B**) LFC of the four clusters with the most significant expression profiles. (**C**) Gene Ontology (GO) overrepresentation analysis of the clusters. (**D**) Gene set enrichment analysis (GSEA) of GO terms for BTP-001 (3 µM) and R11 (1 µM) 10 min after treatment. (**E**) The LFC and *P*-value of selected genes after treatment with BTP-001 (3 µM) for 10 min as compared to an untreated control. Measured using RNA-sequencing and RT-qPCR.

Hierarchical clustering revealed six groups with distinct gene expression patterns following treatment with low-dose BTP-001 (Fig. 3A and B). Clusters 1 and 2 peaked in log_2_ fold change at 10 min, with cluster 2 showing a more pronounced response (Fig. 3A). Both clusters returned to basal expression levels by 50 min. Overrepresentation analysis (ORA) of enriched gene ontology (GO) terms identified genes in clusters 1 and 2 as primarily associated with iron transport and siderophore processes (Fig. 3C). Cluster 3 showed a sustained increase in expression between 25 and 50 min, with enrichment for genes involved in colanic acid metabolism and exopolysaccharide biosynthesis (Fig. 3A through C). Cluster 4 exhibited a more complex expression pattern with peaks at 1 and 50 min. This cluster was enriched for genes involved in polysaccharide metabolism and oxidative stress (Fig. 3A through C). Clusters 1–4 highlight the rapid effect of BTP-001 in *E. coli*, which corresponds with previous results from *S. aureus* and *S. epidermidis* (8, 12).

Interestingly, cluster 5 displayed little change compared to the untreated control, except for a small increase at 25 min. A GO overrepresentation analysis indicated that a metabolic shift occurred at 25 min. This response was not seen in minimal media and might therefore be a response specific to rich media (data not shown). Finally, cluster 6, the largest group, contained genes with no significant change in expression, indicating minimal impact on core metabolic processes like carbohydrate metabolism and the TCA cycle (Fig. 3A and C). In hindsight, the low dose of BTP-001 was likely insufficient to detect all of the various effects observed at doses that affect growth.

For the time-series data, clustering of the log_2_ fold change over time was used to identify significant GO terms. For BTP-001 3 µM and R11, only one time point was sampled. Therefore, gene set enrichment analysis (GSEA), which considers the log_2_ fold change of all genes at the sample time point (10 min), was used to identify significant GO terms. GSEA revealed that the high dose of BTP-001 (3 µM) elicited similar responses as the low dose at 10 min, but with stronger log_2_ fold change changes and more DEGs annotated to the same GO terms. Additionally, the higher dose downregulated genes involved in translation and chemotaxis (Fig. 3D). GSEA suggests that the observed effects of BTP-001 on iron uptake processes might be due to the presence of R11.

To validate the RNA-seq findings, RT-qPCR was employed to evaluate the expression of DEGs with high log_2_ fold change following exposure to BTP-001 (3 µM; Fig. 3E). All selected genes exhibited expression trends consistent with the RNA-seq data, although with a lower log_2_ fold change. The RNA-seq data originated from bioreactor cultures, while the RT-qPCR data were derived from shake flask cultures; thus, the observed differences in log_2_ fold change may be attributed to variations in the cultivation conditions and/or the sensitivity of the different methods. Bioreactors offer a more tightly controlled environment compared to shake flasks, potentially leading to a more pronounced effect.

**Fig 4:**
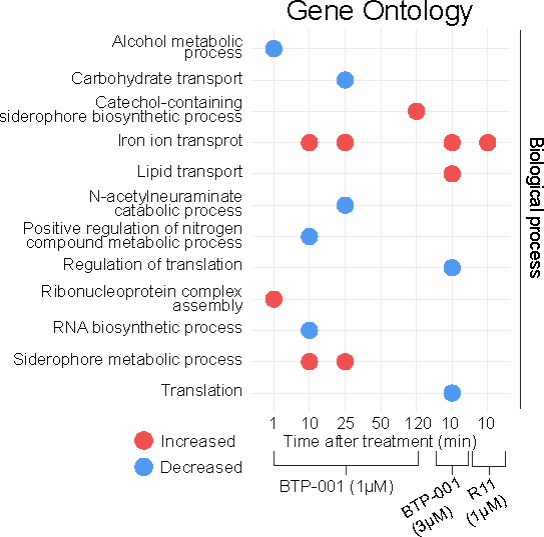
The protein enrichment data from the MIB assay were analyzed using gene set enrichment analysis (GSEA), revealing significant gene ontology terms in *E. coli* following treatment with BTP-001 (1 µM and 3 µM) or R11 (1 µM).

Based on the protein enrichment data from the MIB assay, we can infer the processes that BTP-001 activates or deactivates. The changes in the protein pool following BTP-001 and R11 treatment were analyzed using GSEA (Fig. 4). BTP-001 treatment resulted in increased activation of proteins involved in iron import and siderophore biosynthesis, mirroring the upregulation of corresponding processes in the gene expression data. As seen by both the total number of DEGs/DEPs and further analyses (Fig. 2 to 4), the MIB assay detected more processes that were downregulated in response to BTP-001 than RNA-seq, probably due to effects occurring independently from transcription, e.g., inhibition of translation or post-translation modifications (PTMs). This further highlights the importance of including more than one level of omics analysis.

Collectively, the transcriptomic and protein enrichment analyses indicate that BTP-001 impacts membrane-associated processes, as anticipated for a cationic peptide. While some membrane effects were expected, this analysis differentiated between R11 responses and those specifically attributable to the β-clamp or other targets of BTP-001.

### BTP-001 binding to the β-clamp mediates antimutagenic activity

**Fig 5:**
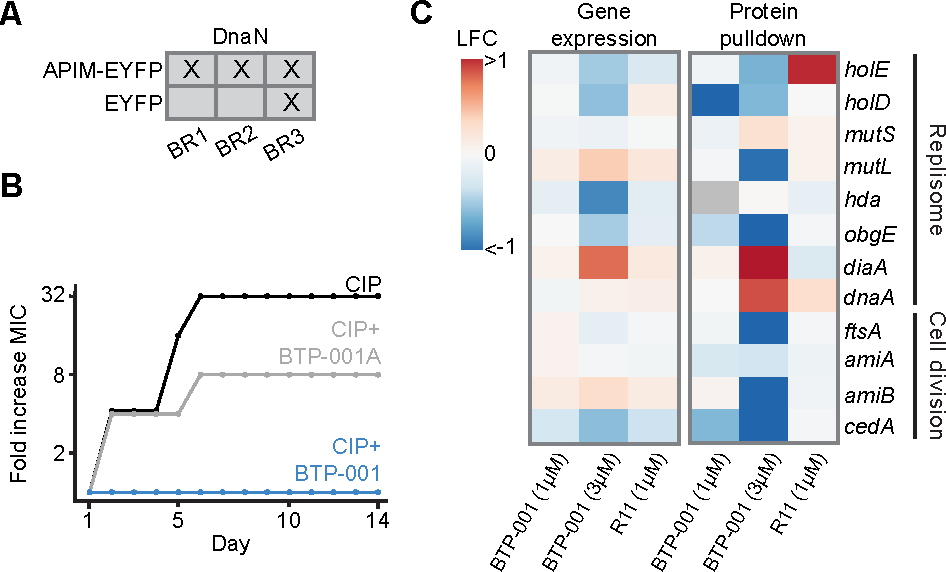
BTP-001 inhibits DNA replication and translation synthesis by binding to the β-clamp in *E. coli***.** (**A**) X: DnaN pulled down during immunoprecipitation. BR: biological replicate. (**B**) The fold change in the ciprofloxacin (CIP) MIC for serial passage of *E. coli* exposed to CIP, CIP + BTP-001, and CIP + BTP-001A. *E. coli* was treated with a fixed concentration of 4 µM BTP-001 or BTP-001A, while CIP concentrations ranged from 0.012 to 0.048 µM depending on the level of resistance. Log_10_ *y*-axis. One representative BR out of three experiments with same trends is shown, and the remaining two BRs are shown in Fig. S1. (**C**) Gene expression and protein pull-down by the MIB assay 10 min after BTP-001 treatment. Gray indicates no data.

APIM (RWLVK) has previously been shown to bind the β-clamp, resulting in inhibition of replication and TLS (6). Here, we further verified the APIM-β-clamp interaction with immunoprecipitation (IP) of APIM fused to enhanced yellow fluorescent protein (EYFP; APIM-EYFP) in *E. coli*. The β-clamp, also known as DnaN, was pulled down in three biological replicates of *E. coli* overexpressing APIM-EYFP, as compared to only one biological replicate in the EYFP-only controls (Fig. 5A).

APIM-containing peptides, including BTP-001, reduced the spontaneous and UV-induced mutation frequency in *E. coli* (Rif^R^ assay), likely due to inhibition of Pol V (UmuD´_2_C*), the main TLS polymerase in *E. coli* (6). This study aims to demonstrate that BTP-001 reduces the resistance development to ciprofloxacin (CIP) using a serial passage experiment. Since TLS promotes increased resistance development through mutagenesis, this will also indirectly verify APIM’s ability to inhibit TLS. BTP-001 was compared to BTP-001A, which contains a mutated APIM, RALVK, leading to reduced affinity toward the β-clamp (6) and its eukaryotic homolog, PCNA (24). Therefore, BTP-001A can serve as a control for the importance of APIM binding to the β-clamp for reduced TLS. The results revealed no resistance development for CIP + BTP-001, while CIP and CIP + BTP-001A experienced a 32 and 8-fold increase in MIC, respectively (Fig. 5B). This was further supported by two additional biological replicates showing a similar trend of lower resistance development for CIP + BTP-001 compared to CIP and CIP + BTP-001A (Fig. S1). These findings strongly support the critical role of APIM binding to the β-clamp in mediating the antimutagenic properties of BTP-001.

The β-clamp functions as an indispensable scaffold during replication, interacting with several DNA replication proteins, including DNA polymerases, ligase, mismatch repair proteins (MutS and MutL), and Hda, which mediates the interaction between the β-clamp and the replication initiator protein DnaA. Protein enrichment data from the MIB assay for both the high- and low-dose BTP-001, alongside the high-dose transcriptome data, revealed a decrease in these DNA replication proteins following BTP-001 exposure for 10 min (Fig. 5C). This suggests that BTP-001 may trigger either deactivation or degradation of these proteins, even at the low dose, which did not impede bacterial growth (Fig. 1A and B). The β-clamp is known to function in replication initiation by inactivating DnaA after formation of the replisome [reviewed in (25)]. Therefore, BTP-001 might inhibit the β-clamp-mediated inactivation of DnaA, resulting in the observed enrichment of activated DnaA and its interaction partner DiaA (Fig. 5C).

BTP-001 led to the decreased pulldown of several cell division proteins in the MIB assay, despite unaltered gene expression levels, suggesting deactivation or degradation. For example, the highest dose of BTP-001 reduced the pulldown of FtsA, a protein essential for septum formation (Fig. 5C). Similarly, pulldown of the proteins AmiA/AmiB (septum cleavage) and CedA (transcriptional activator of cell division) was observed in the control but not after BTP-001 treatment. In line with these data, BTP-001 was previously shown to reduce the pulldown of FtsA in *S. aureus*, and interactome studies indicated that APIM-EYFP and the β-clamp were in the same complex as FtsA and FtsZ, respectively (12). Collectively, these results suggest that BTP-001 binding to the β-clamp disrupts cell division, potentially due to a noncanonical role of the β-clamp as a scaffold for these proteins.

### R11 mediates BTP-001 uptake via an iron transport system

**Fig 6:**
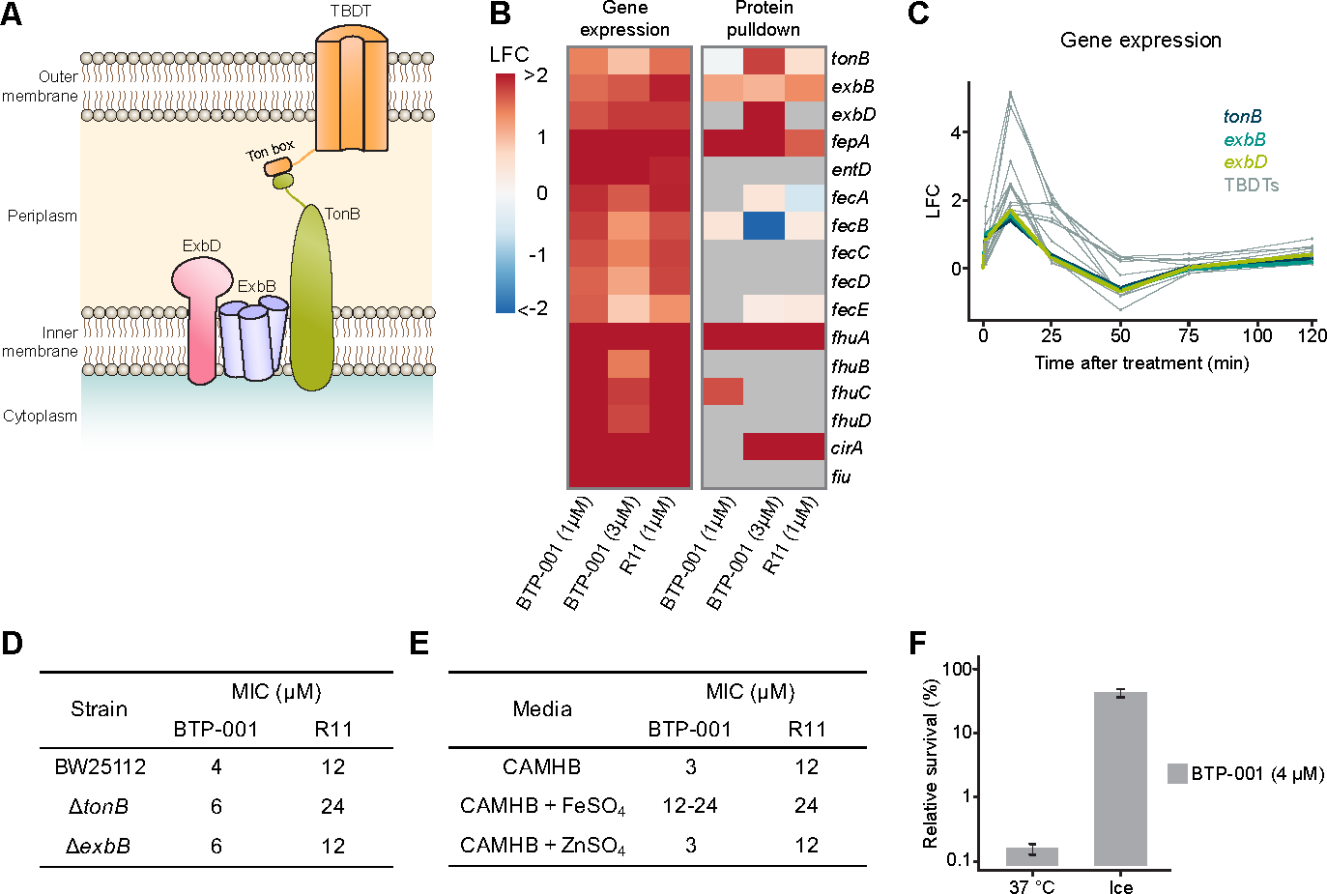
Iron import systems are activated upon BTP-001 treatment. (**A**) The TonB import system. TBDT: TonB-dependent transporter. (**B**) Gene expression and protein pulldown log_2_ fold change (LFC) following treatment of *E. coli* with BTP-001 and R11 relative to an untreated control. (**C**) Time series LFC of gene expression following treatment with BTP-001 (1 µM). (**D**) MIC of *E. coli* BW25113 wild type (WT) and the *E. coli* BW25113 single-gene knockouts Δ*tonB* and Δ*exbB* from the Keio collection for BTP-001 and R11. (**E**) MIC of *E. coli* MG1655 for BTP-001 and R11 with no supplementation or with FeSO_4_ or ZnSO_4_ (80 µM) supplementation. (**F**) Survival, measured as CFU/mL, after treatment with BTP-001 (4 µM) at 37°C or on ice. Displayed on a log_10_ scale relative to the untreated control.

Bacteriocins, antimicrobial peptides produced by bacteria, often hijack iron import systems to cross the cell membrane (26). The bacteriocin colicin B uses the TonB system, which includes an outer membrane (OM) TonB-dependent transporter (TBDT), in addition to the inner membrane (IM) proteins TonB, ExbB, and ExbD, as illustrated in Fig. 6A (27). *E. coli* possesses several known TBDTs [reviewed in (28)]. Interestingly, both BTP-001 and R11 triggered increased expression of genes regulating the TonB uptake system, and the MIB assay indicated activation of the corresponding proteins (Fig. 6B). For the low dose, expression of these genes peaked at 10 min, with expression levels returning to baseline levels within 50 min (Fig. 5C). This suggests a link between iron import and transport of BTP-001 across the cell membrane. The transient nature of this expression suggests that once the initial transport and uptake of BTP-001 have occurred, iron transporters are no longer needed, and the cell downregulates expression. Nevertheless, we detected an increase in the MIC for BTP-001 in Δ*tonB* and Δ*exbB* strains (Fig. 6D).

All iron import DEGs are regulated by the transcriptional repressor Fur, which dissociates from the DNA under iron-limiting conditions. The relationship between iron and BTP-001 uptake is further supported by an increase in the MIC of both BTP-001 and R11 in *E. coli* following supplementation of the growth medium with FeSO₄ (Fig. 6E). To differentiate between the effects of iron itself and the ionic charges (Fe^2+^/SO_4_^2-^), we also tested ZnSO_4_, which had no effect on the MIC of BTP-001. The iron effect was observed in both complex and minimal media for BTP-001 as well as in *S. aureus* (data not shown). Overall, this strongly suggests that iron interferes with the processes needed to transport BTP-001 across bacterial cell membranes. Perhaps higher iron concentrations saturate iron import systems, thereby preventing BTP-001 from “hitching a ride.” Importantly, the human body tightly regulates the levels of free iron to prevent bacterial growth; therefore, increased MIC in the presence of iron will most likely not be a problem *in vivo*.

BTP-001 was previously proposed to cross the cell membrane via an energy-dependent process (7). To assess whether reduced ATP production could affect BTP-001 uptake and its associated lethality, the survival (CFU/mL) of *E. coli* was determined after exposure to BTP-001 (4 µM) either on ice or at 37°C. After 10 min of exposure, the cells were washed and incubated for 30 min at 37°C. When exposed to BTP-001 on ice, a 57% reduction in CFU/mL was observed compared to a reduction of almost 99.8% when exposed at 37°C (Fig. 6F). This demonstrates a more than 200-fold reduction in the bactericidal effect of BTP-001 on ice compared to at 37°C. This is likely due to reduced uptake. If BTP-001 was taken up by the cells while on ice, but its lethal effects were energy-dependent, then the 30 min incubation at 37°C after treatment would have resulted in cell death. These results suggest that the uptake of BTP-001 is dependent on active transport. However, these results require further validation.

### BTP-001 affects pulldown of ribosomal proteins in the MIB assay and reduces translation

**Fig 7:**
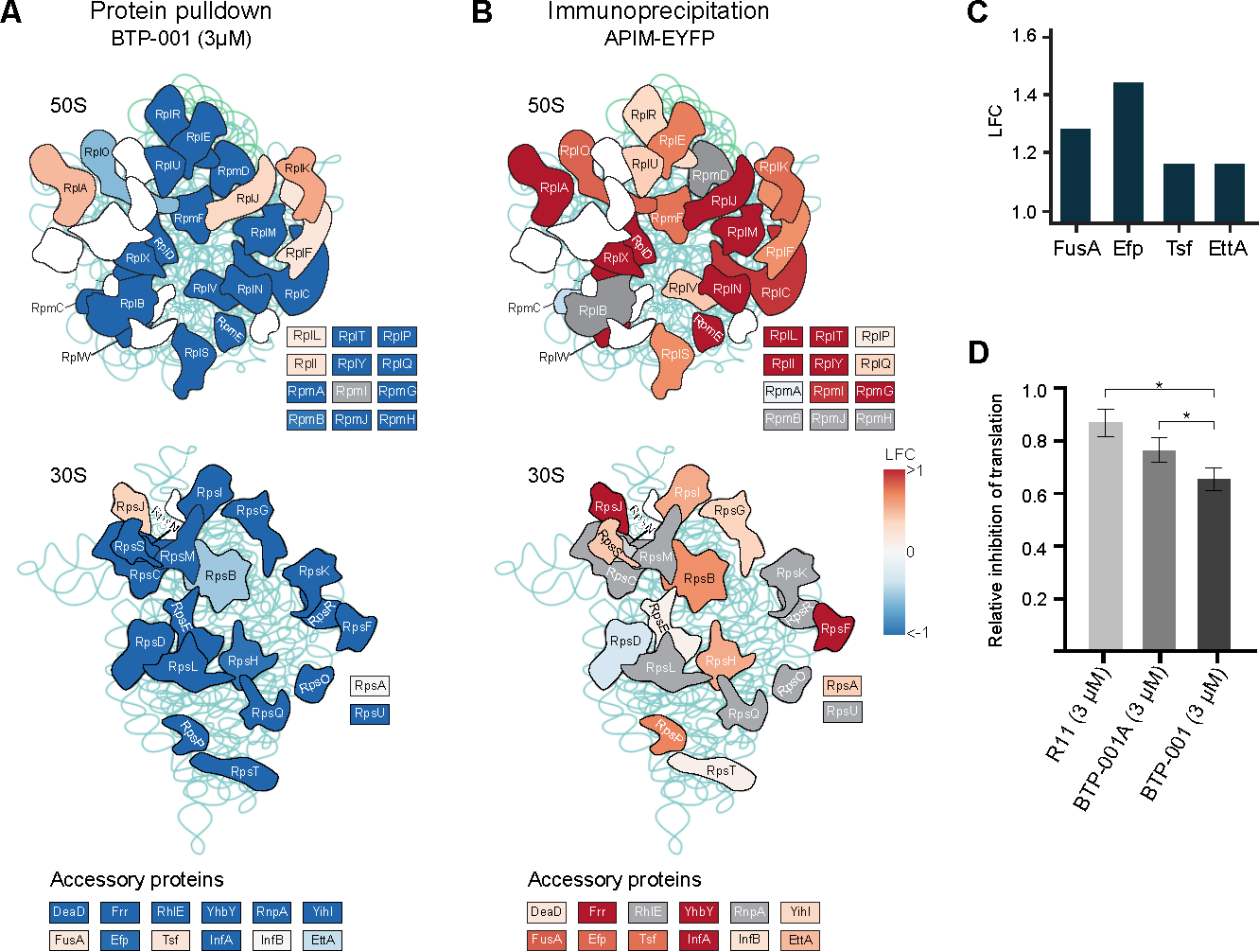
BTP-001 affects pull-down of ribosomal proteins and translation. (**A**) Log_2_ fold change (LFC) of protein pull-down using the MIB assay for ribosomal proteins in the 50S (top) and 30S (bottom) subunits 10 min after BTP-001 (3 µM) treatment. LFC is shown relative to the untreated control. Accessory ribosomal proteins are listed at the bottom. (**B**) Immunoprecipitation of *E. coli* overexpressing APIM-EYFP. LFC is shown as APIM-EYFP relative to EYFP-only control. Raw data are shown in Table S1. (**A,B**) Gray indicates that the protein was not detected. The 50S and 30S subunit structures are from KEGG and are based on the *Haloarcula marismortui* and *Thermus aquaticus* ribosomes, respectively (29). A common structural core is shared by most species. White with no name indicates that the protein is not part of the *E. coli* ribosome. (**C**) LFC in protein pull-down using His-tagged β-clamp relative to a His-tag only control. Raw data are shown in Table S2. (**D**) The effects of BTP-001, BTP-001A, and R11 (3 µM) on luciferase expression during *in vitro* translation. *P < 0.05.

Bacterial ribosomes are composed of around 35% protein and 65% ribosomal RNA. Decreased pulldown of ribosomal and accessory proteins in the MIB assay was the most prominent effect on the proteome after BTP-001 treatment (Fig. 7A). However, the corresponding genes were not differentially expressed, indicating degradation or deactivation of these proteins (Fig. S2). Notably, the low dose did not impact ribosomal protein pulldown, likely because the dose was too low to affect the growth. These results are consistent with previous observations in *S. aureus*, in which a high dose of BTP-001 also resulted in decreased pulldown of ribosomal proteins (12).

Bacterial cells tightly regulate ribosome synthesis and degradation to maintain protein homeostasis. This balance is vital as ribosomal proteins constitute about 25% of the total protein synthesis in bacteria. While ribosomes are generally stable, degradation can occur during starvation and after treatment with certain antibiotics as translational control is a rapid and important stress response in all organisms (30, 31). Several factors may contribute to the deactivation or degradation of ribosomes observed after BTP-001 treatment, including the following: (i) BTP-001 binds the β-clamp via APIM, leading to stalled replication. In bacteria, transcription and translation are concurrent processes, and therefore stalled replication by BTP-001 may lead to more collisions with transcription and thus also disrupt translation; (ii) increased stress caused by BTP-001 may trigger ribosome inactivation and degradation, to conserve energy for stress responses; (iii) as BTP-001 interacts with the cell membrane, this may cause cytoplasmic ionic imbalances, potentially disrupting the ribosome structure and promoting degradation due to the dependence on Mg^2+^ to maintain structural integrity (31); (iv) the β-clamp may play a scaffolding role in ribosomes. The latter is supported by the fact that several ribosomal proteins with significantly reduced pulldown in the MIB assay are also immunoprecipitated with APIM-EYFP (Fig. 7A and B; Table S1), suggesting that the reduced pulldown is due to disruption of ribosomal complexes by BTP-001. The APIM-EYFP interaction with ribosomal proteins could be direct via APIM or indirect via the β-clamp. The latter is supported by the increased pull-down of the elongation factors FusA (EF-G), Efp (EF-P), and Tsf (EF-Ts) and the translation factor EttA by the His-tagged β-clamp as compared to the His-tag only control (Fig. 7C; Table S2). To explore how targeting the β-clamp affects translation, we compared the effects of BTP-001, BTP-001A, and R11 in an *in vitro* translation assay (17). We found that all peptides reduced the translation efficacy, potentially caused by a cationic peptide effect on RNA and/or ribosomes. However, BTP-001 was more efficient than the similar peptide with lower affinity to the β-clamp, BTP-001A (6), while the peptide representing the cell-penetrating part, R11, had the weakest effect (Fig. 7D). The stronger translation inhibition by BTP-001 compared to BTP-001A might be due to stronger binding of APIM to the β-clamp, providing additional support for a potential scaffolding role of the β-clamp in translation.

### Cell envelope stress induced ROS mediate the rapid bactericidal effect of BTP-001

**Fig 8:**
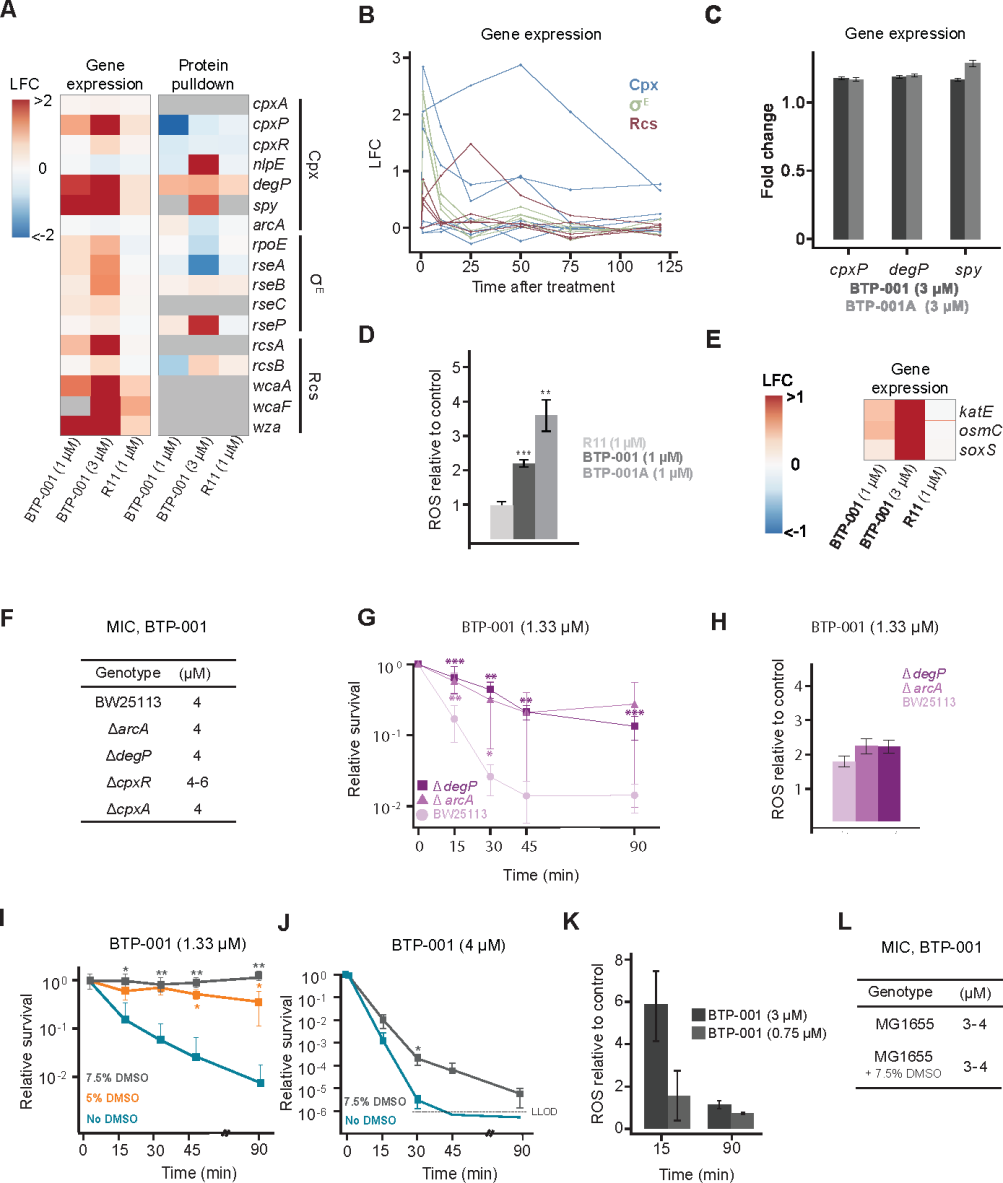
BTP-001 triggers the Cpx response, which induces rapid reactive oxygen species (ROS)-dependent killing of *E. coli***.** (**A**) Gene expression and protein pull-down 10 min after treatment with BTP-001 and R11 relative to an untreated control. LFC: log_2_ fold change. (**B**) LFC of gene expression from 1 to 120 min after treatment with BTP-001 (1 µM). Genes are categorized according to the specific cell envelope stress response they are involved in. (**C**) Fold change of the gene expression measured using RT-qPCR after treatment with BTP-001 (3 µM) and BTP-001A (3 µM). (**D**) ROS produced after treatment with BTP-001, BTP-001A, and R11 (all 1 µM) compared to an untreated control. (**E**) LFC of gene expression 10 min after treatment with BTP-001 and R11. (**F**) MIC determination for BTP-001 in *E. coli* BW25112 wild type (WT) and Keio collection mutants. (**G**) Relative survival based on CFU/mL count after treatment of *E. coli* BW25113 (WT), Δ*degP*, and Δ*arcA* with BTP-001 (1.33 µM) in time-kill assays. (**H**) Relative ROS after treatment of *E. coli* BW25113 (WT), Δ*degP*, and Δ*arcA* with BTP-001 (1.33 µM). (**I, J**) Relative survival based on CFU/mL after treatment with BTP-001 (1.33 or 4 µM) supplemented with the ROS scavenger DMSO in time-kill assays. LLOD: lower limit of detection. (**K**) Relative ROS at 15 and 90 min after treatment with BTP-001 (3 or 0.75 µM). (**L**) MIC determination for BTP-001 in MG1655 with and without presence of 7.5% DMSO (**D, G, I, J**) *P ≤ 0.05, **P < 0.01, ***P < 0.001, and ****P < 0.0001.

Cell envelope damage induces finely tuned mechanisms to maintain membrane integrity and promote repair. Combined gene expression and MIB protein enrichment analysis revealed that both low and high doses of BTP-001 resulted in upregulation and activation of multiple genes and proteins involved in cell envelope repair and biogenesis. These included genes and proteins involved in the σ^E^, Cpx, and Rcs cell envelope stress response pathways (Fig. 8A). Activation of these pathways was rapid, with peak expression observed at 1 min with the low dose, followed by a rapid decline in the expression for most genes (Fig. 8B). Furthermore, the Rcs response upregulates genes involved in capsule biosynthesis (*wcaA, wcaF,* and *wza*; Fig. 8A). This process can increase resistance by reducing the transport of AMPs across the membrane (1). Interestingly, R11-treated *E. coli* displayed no cell envelope stress, implying that this is a full-length peptide effect.

PCNA, the eukaryotic homolog to the β-clamp, has recently been shown to have scaffolding roles outside DNA replication, which are important for regulating signaling and metabolism during cellular stress. This is mediated through the interaction of PCNA with APIM in APIM-containing proteins and is blocked by APIM-containing peptides (32). Therefore, BTP-001 might also trigger stress responses by blocking potential noncanonical roles of the β-clamp. To determine if cell envelope stress is caused by APIM binding to the β-clamp, we used RT-qPCR to compare the expression of cell envelope stress genes (*cpxP*, *degP,* and *spy*) in *E. coli* treated with BTP-001 and BTP-001A, the latter containing the mutated APIM with lower affinity for the β-clamp. Both triggered expressions of cell envelope stress response genes (Fig. 8C). These findings suggest that the cell envelope stress caused by BTP-001 may be attributed to the full-length cationic nature or structure, rather than interactions with the β-clamp.

Independent of the primary target, antibiotic-induced lethality is hypothesized to be linked to increased ROS (33, 34). Previously, we showed that BTP-001 in *S. aureus* induced a dose-dependent increase in intracellular ROS as well as decreased activation of the redox sensing transcriptional repressor Rex (12). Here, we observed that BTP-001A yields higher ROS production in *E. coli* compared to BTP-001 (Fig. 8D), even though the MIC of BTP-001A is higher than that of BTP-001 (>2×; Table 1 ). In contrast, R11 induced significantly less ROS. This indicates that ROS, similarly to the cell envelope stress responses, is not triggered by R11-mediated translocation across the cell envelope, but by the full-length peptide, and that ROS induction is independent of the β-clamp interaction. This view is further strengthened by an upregulation of genes encoding ROS-detoxifying enzymes (*katE* and *osmC*) and the transcriptional activator *soxS*, a regulator of superoxide stress genes, only in response to BTP-001 and not R11 (Fig. 8E).

Some studies have proposed that the Cpx response is responsible for aminoglycoside-induced cell death via ROS (35, 36). During activation of Cpx, ArcA might become phosphorylated by CpxA, leading to transcriptional activation of several respiratory genes. Thus, ArcA is proposed to link the Cpx pathway to respiration. This could potentially disrupt the respiratory chain and induce ROS. To assess the role of the Cpx response in BTP-001’s antibacterial activity, we measured growth inhibition by MIC in Δ*arcA*, Δ*degP*, Δ*cpxR*, Δ*cpxA,* and WT strains from the Keio collection (14) and compared this with bactericidal effects observed in time-kill assays using Δ*arcA*, Δ*degP,* and WT. MICs for Δ*arcA,* Δ*degP,* and Δ*cpxA* were the same as for WT, while Δ*cpxR* had a slightly higher MIC (Fig. 8F). Despite the same MIC as WT, Δ*arcA* and Δ*degP* strains demonstrated significantly higher survival rates the first 30 min in the time-kill assays following exposure to BTP-001 (Fig. 8G). However, the ROS levels in these two mutant strains after treatment with BTP-001 were not significantly different from WT after 30 minutes (Fig. 8H).

To further explore the correlation between the rapid bactericidal effect and ROS, we included DMSO as an ROS scavenger as it is previously shown to protect bacteria from ROS-induced lethality (37, 38). The addition of DMSO reduced BTP-001-mediated lethality at both sub-MIC and MIC doses (Fig. 8I and J). Further, we found that the ROS induction by BTP-001 was transient and mainly gone after 90 min (Fig. 8K) and that DMSO had no effects on MIC (Fig. 8L). Thus, our data indicate that ROS is important for the rapid bactericidal activity of BTP-001 but does not solely mediate its antibacterial activity. This is in line with previous studies demonstrating a role of ROS in rapid antibiotic-induced killing but not necessarily in overall growth inhibition (37–39). MIC is a less sensitive method to assess the contribution of ROS in bactericidal activity as it (i) reflects the growth inhibition rather than killing; (ii) measures the slower effects while ROS is mainly associated with rapid lethality; (iii) uses twofold dilution steps that cannot detect small changes in viability. Our data suggest that the MIC of BTP-001 is primarily determined by its interaction with its primary target, the β−clamp, and inhibition of replication, rather than ROS induction. This is also supported by the omics data and resistance development assay showing that BTP-001 treatment affects replication, TLS, and translation. In addition to the unchanged MIC after DMSO treatment, the fact that the MIC of BTP-001A is >2 fold higher than that of BTP-001 (Table 1), despite a higher ROS-inducing capacity (Fig. 8D), supports the central role of β-clamp inhibition in BTP-001’s antibacterial activity.

### Conclusion

This study revealed a multifaceted mode of action for the APIM-containing peptide BTP-001, a promising antibacterial agent. BTP-001 exhibits dual activity, targeting the replisome, TLS, and potential translation regulation through APIM-dependent interaction with the β-clamp. Additionally, the full-length peptide shows significant effects on cell envelope stress responses, which may be responsible for BTP-001’s rapid bactericidal effect via ROS production. Finally, the energy-dependent uptake of BTP-001 facilitated by R11 underscores the importance of investigating peptide transport mechanisms. This knowledge is crucial for optimizing future antimicrobial drug candidates. Overall, these findings significantly enhance our understanding of BTP-001 and pave the way for further investigation into its potential as a therapeutic agent.

## Data Availability

Raw RNA-seq data and the corresponding count file have been deposited together with raw data files on the NCBI Gene Expression Omnibus (GEO) under the accession number GSE276254 (40). The proteomics data have been deposited to the ProteomeXchange Consortium via the PRIDE partner repository with the data set identifier PXD055475 (41).

## SUPPLEMENTAL MATERIAL

Supplemental material msphere.00068-25-s0001.pdf Supplemental figures and tables.

ASM does not own the copyrights to Supplemental Material that may be linked to, or accessed through, an article. The authors have granted ASM a non-exclusive, world-wide license to publish the Supplemental Material files. Please contact the corresponding author directly for reuse.
