# Supplementary material for: A dual-mechanism antimicrobial peptide with antimutagenic activity targets the replisome and induces cell envelope stress
Source: mSphere. 2025 Aug 29;10(9):e00068-25. doi: 10.1128/msphere.00068-25 (PMC12482183; doi:10.1128/msphere.00068-25)
Supplement: Supplemental material — Supplemental figures and tables. [file msphere.00068-25-s0001.pdf]

Supplementary Figures

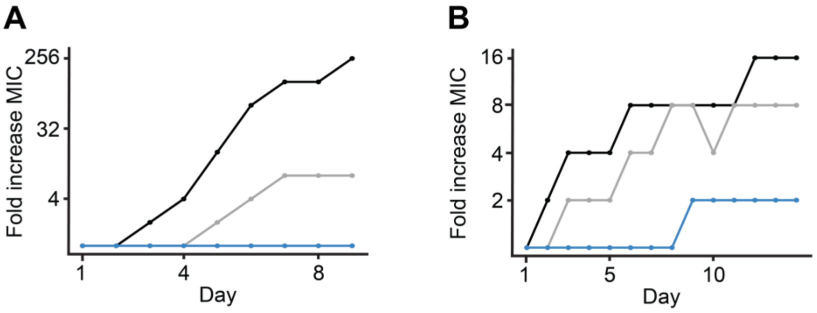

Supplementary Figure S1: Two additional biological replicas related to Figure 5B. The fold change in the ciprofloxacin (CIP) MIC for serial passage of *E. coli* MG1655 exposed to CIP, CIP + BTP-001, and CIP + BTP-001A. *E. coli* was treated with a fixed concentration of 4 µM BTP-001 or BTP-001A, while CIP concentrations ranged from 0.012 to 0.048 µM depending on the level of resistance. Log10 y-axis.

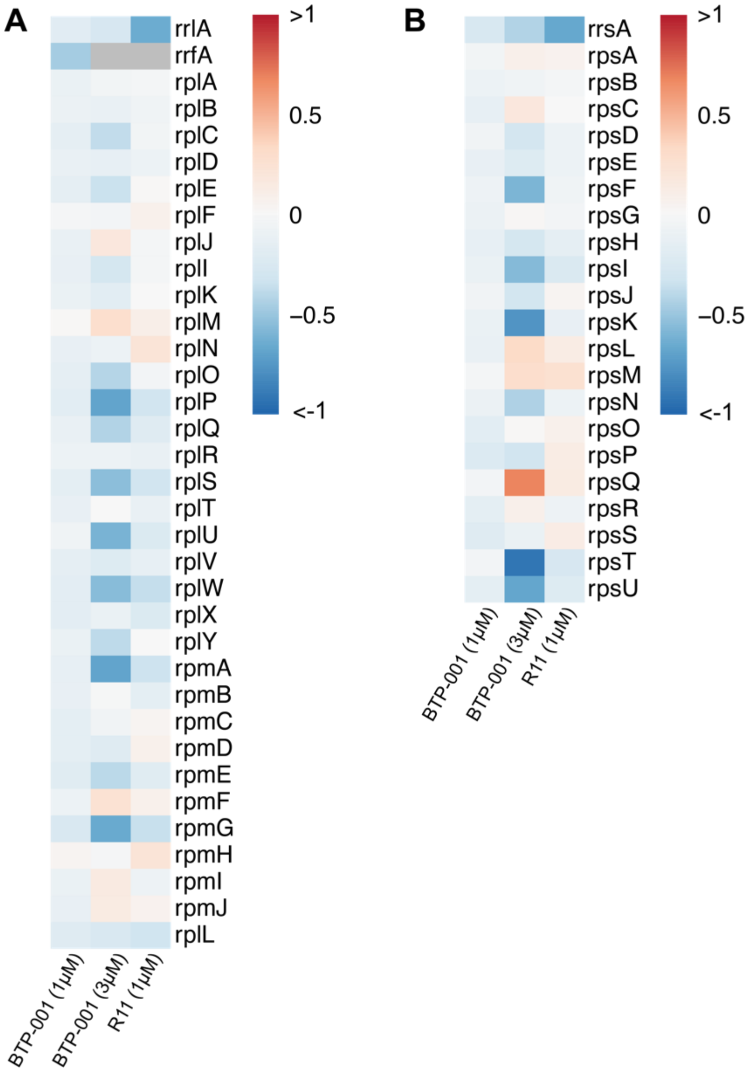

Supplementary Figure S2: The log_2 fold-change in gene expression of ribosomal proteins in the (A) 50S and (B) 30S subunits. Related to Figure 7.

Supplementary Table S1: The log_2 label free quanitifaction intensities of ribosomal proteins from Figure 7B pulled down with APIM-EYFP and EYFP as a measure of relative protein abundance, as calcuated in MaxQuant from the raw data files. *Italics* indicates imputed values. **Bold** indicates a p-value below 0.1 using a two-sided Student's *t*- test.

Protein	APIM-EYFP Biological replicate			EYFP-only Biological replicate			Log_2 fold-change	P-value
	1	2	3	1	2	3		
DeaD	23.65	24.18	24.92	24.05	23.69	24.39	0.21	0.655
Efp	23.04	23.65	24.90	23.09	23.28	22.84	0.79	0.282
EttA	19.72	22.28	23.79	21.45	21.11	21.36	0.62	0.653
Frr	23.98	24.66	25.58	23.69	23.83	23.83	0.96	0.174
FusA	25.65	26.56	27.51	25.77	25.84	25.84	0.76	0.294
InfA	22.14	23.07	25.46	22.55	22.58	22.62	0.97	0.429
InfB	24.22	24.43	24.66	24.20	23.72	24.44	0.32	0.283
RplA	26.48	27.69	28.12	26.37	25.69	26.49	1.25	0.109
RplC	26.22	27.44	27.58	26.16	26.35	25.91	0.94	0.153
RplD	25.97	26.99	27.79	25.66	25.38	26.07	1.21	0.135
RplE	27.77	27.81	27.61	27.03	27.22	27.34	0.53	**0.011**
RplF	26.60	27.45	27.82	26.65	26.73	26.42	0.69	0.191
RplI	26.02	26.68	27.63	25.57	24.84	25.52	1.47	**0.069**
RplJ	25.52	26.93	27.98	25.41	26.02	25.56	1.15	0.245
RplK	26.82	27.30	28.03	26.63	25.98	25.77	1.26	**0.050**
RplL	27.78	28.35	28.66	27.28	27.63	27.46	0.81	**0.073**
RplM	25.86	25.82	25.83	24.76	24.98	24.76	1.00	**0.004**
RplN	27.90	27.77	27.38	26.49	26.66	26.55	1.12	**0.013**
RplO	27.24	27.17	27.64	26.56	26.89	26.42	0.73	**0.023**
RplP	26.22	25.77	27.01	26.21	*22.76*	26.24	1.26	0.391
RplQ	27.15	26.88	27.22	26.73	24.76	26.77	1.00	0.270
RplR	27.08	27.03	27.31	26.80	23.46	26.48	1.56	0.280
RplS	26.77	26.23	26.70	25.92	25.28	25.63	0.96	**0.019**
RplT	27.59	27.75	27.71	26.78	25.31	26.79	1.39	0.104
RplU	26.70	26.39	27.68	26.53	26.10	26.28	0.62	0.247
RplV	26.15	25.89	28.36	26.39	24.12	25.91	1.33	0.274
RplW	23.04	24.81	26.03	23.00	23.51	22.93	1.48	0.227
RplX	26.40	27.12	27.86	25.64	26.04	26.05	1.22	**0.090**
RplY	26.03	26.42	26.90	24.95	25.07	24.72	1.54	**0.015**
RpmA	25.47	25.34	25.36	25.48	25.04	24.37	0.43	0.317
RpmC	25.77	23.14	26.08	25.24	21.73	23.55	1.49	0.341
RpmE	*21.41*	23.83	23.87	*21.54*	*22.97*	22.59	0.67	0.517
RpmF	24.22	23.19	24.17	23.13	23.06	22.55	0.95	**0.087**
RpmG	24.55	25.07	25.79	23.93	*23.13*	24.36	1.33	**0.059**
RpmI	22.85	24.09	23.95	22.71	*20.55*	22.81	1.61	0.149
RpsA	25.54	26.01	26.90	25.77	25.80	25.71	0.39	0.431
RpsB	26.44	26.68	27.59	26.26	25.71	26.10	0.88	0.113
RpsD	27.15	27.51	27.60	27.55	27.51	27.36	-0.05	0.747
RpsE	26.90	27.21	27.66	27.17	26.47	26.84	0.43	0.225
RpsF	26.74	26.01	26.57	25.44	24.69	25.07	1.37	**0.011**
RpsG	24.96	25.97	27.53	25.79	25.70	25.48	0.50	0.576
RpsH	26.15	27.09	27.01	26.21	26.46	25.54	0.68	0.171
RpsI	27.30	27.26	27.43	26.80	26.77	26.62	0.60	**0.001**
RpsJ	26.85	27.45	27.97	26.00	26.67	26.26	1.11	**0.054**
RpsP	24.73	25.43	26.44	24.84	24.96	25.18	0.54	0.391
RpsS	26.08	26.63	27.76	26.40	26.23	25.51	0.78	0.260
RpsT	26.12	26.13	26.42	26.13	25.22	25.56	0.59	0.146
Tsf	25.69	26.74	27.35	25.84	26.07	25.81	0.69	0.291
YhbY	*20.72*	21.94	22.68	*21.37*	*23.07*	*22.23*	-0.45	0.586
YihI	19.87	20.52	20.26	19.88	*19.26*	19.72	0.60	**0.087**

Supplementary Table S2: The log_2 label free quanitifaction intensities of ribosomal proteins from Figure 7C pulled down with His-tagged β-clamp and His-tag as a measure of relative protein abundance, as calcuated in MaxQuant from the raw data files. *Italics* indicates imputed values. **Bold** indicates a p-value below 0.1 using a two-sided Student's *t*- test.

Protein	His-tagged β-clamp Biological replicate				His-tag only Biological replicate			Log_2 fold-change	P-value
	1	2	3	4	1	2	3		
FusA	30.88	28.59	28.91	30.65	28.75	28.45	28.26	1.27	0.117
Efp	27.38	25.56	25.99	27.39	24.87	24.73	25.82	1.44	**0.057**
Tsf	29.41	28.04	27.61	29.79	27.66	27.54	27.43	1.16	0.113
EttA	21.31	24.85	26.31	*22.83*	22.13	21.91	23.96	1.16	0.409
